# Providing Insights into the Challenges of Implementing Activity-Based Therapy in Canada: A Comparative Analysis Using Focus Group Interviews with Key Interest Groups

**DOI:** 10.46292/sci23-00022S

**Published:** 2023-11-17

**Authors:** Anita Kaiser, Katherine Chan, James Sessford, Shane McCullum, Peter Athanasopoulos, Chris Rice, Jennifer Leo, Iona MacRitchie, José Zariffa, Kristin E. Musselman

**Affiliations:** 1 Rehabilitation Sciences Institute, Temerty Faculty of Medicine, University of Toronto, Toronto, ON, Canada;; 2 KITE-Toronto Rehabilitation Institute, University Health Network, Toronto, ON, Canada;; 3 Canadian Spinal Research Organization, Richmond Hill, ON, Canada;; 4 Stan Cassidy Centre for Rehabilitation, Horizon Health Network, Fredericton, NB, Canada;; 5 Spinal Cord Injury Ontario, Toronto, ON, Canada;; 6 The Steadward Centre for Personal & Physical Achievement, University of Alberta, Edmonton, AB, Canada;; 7 Toronto Rehabilitation Institute, University Health Network, Toronto, ON, Canada;; 8 Institute of Biomedical Engineering, University of Toronto, Toronto, ON, Canada;; 9 Edward S. Rogers Sr. Department of Electrical and Computer Engineering, University of Toronto, Toronto, ON, Canada;; 10 Department of Physical Therapy, Temerty Faculty of Medicine, University of Toronto, Toronto, ON, Canada

**Keywords:** exercise therapy, focus groups, qualitative research, spinal cord injury

## Abstract

**Background:**

Activity-based therapy (ABT) has emerged as a therapeutic approach that may promote neurorecovery and reduce secondary complications in people living with spinal cord injury or disease (SCI/D). In spite of the numerous health benefits, adoption of ABT into practice has been limited across the Canadian care continuum.

**Objectives:**

This study aimed to understand the challenges of implementing ABT in Canada for people living with SCI/D through the perspectives of key interest groups.

**Methods:**

Researchers, hospital therapists, community trainers, administrators, persons living with SCI/D, and advocates, funders, and policy experts who had knowledge of and/or experience with ABT participated in focus group interviews to share their perspectives on the barriers to ABT practice. Interviews were analyzed using conventional content analysis followed by a comparative analysis across groups.

**Results:**

The 48 participants identified six key challenges: (1) challenge of gaps in knowledge/training, (2) challenge of standardizing ABT, (3) challenge of determining the optimal timing of ABT, (4) challenge of defining, characterizing, and achieving high dosage and intensity, (5) challenge of funding ABT, and (6) challenge of measuring participation and performance in ABT. A comparative analysis found some challenges were emphasized by certain groups, such as the cost of ABT for persons with SCI/D, lack of education and training in ABT for therapists and trainers, minimal evidence to develop guidelines for researchers and advocates, and funding ABT programs for administrators.

**Conclusion:**

Participants highlighted several challenges that limit ABT practice. Strategies to address these challenges will support successful implementation of ABT in Canada.

## Introduction

Neurorehabilitation has evolved over recent years with the introduction of therapies that target neuromuscular activation below the level of injury. These therapies, known as activity-based therapies (ABT),^1^ involve intensive practice of task-specific movements with the goals of promoting neurorecovery, reducing secondary complications, and improving emotional well-being and overall quality of life for persons living with spinal cord injury or disease (SCI/D).^2^-^7^ ABT may be practiced with or without the aid of technology in various settings across the continuum of care and injury trajectory.^7^-^10^ Despite the versatility and potential health benefits of ABT, implementation in Canada has proved challenging.^7^-^9^

With 86,000 Canadians living with SCI/D and nearly 1400 new cases each year, the staggering $2.7 billion in annual healthcare costs creates a toll on the Canadian healthcare system.^11^ The need for rehabilitation, like ABT, to be available to all individuals with SCI/D, in all settings, at any point in their life course is paramount not only to reducing the burden on the healthcare system^12^ but also to improving the lives of persons living with SCI/D. In response to the state of SCI/D in Canada, a diverse group of 39 individuals from across Canada with an interest in ABT met in 2019 to understand the current situation of ABT in this country and to outline priorities going forward to advance research and clinical care over the next 5 years.^13^ The group included researchers, healthcare administrators, frontline clinicians, funders of SCI research, health policy experts, and persons with SCI/D. Together they formed the Canadian ABT Community of Practice (ABT CoP) with a goal to increase the access to and quality of ABT in Canada for individuals living with SCI/D.

This study allowed us to follow-up on the priorities identified by the ABT CoP by having an in-depth discussion that was focused on key challenges with specific groups of people who have an interest in ABT. Using a comparative analysis approach with multiple perspectives allows researchers to investigate different viewpoints of a specific phenomenon, in this case ABT.^14^ The advantage to using this approach is the ability to highlight differences within and between groups, identify existing barriers specific to only one group, or identify challenges that exist between groups.^14^ Successful strategies toward the implementation of ABT in Canada requires the input of all applicable members to identify existing challenges. As such, the purpose of this study was to understand and compare the perspectives of key interest groups on the challenges of implementing ABT in Canada for people living with SCI/D.

## Methods

This descriptive qualitative study employed focus group meetings with key interest groups. These focus group meetings also explored participants’ perspectives on tracking ABT activities and parameters, with these results summarized in Kaiser et al.^15^ The study received ethical approval from the Research Ethics Board of the University Health Network (REB Protocol #19-6154). Reporting of study details was guided by the Standards for Reporting Qualitative Research (SRQR).^16^

### Participants

Individuals from six different groups were recruited through a national poster campaign, the ABT CoP, and snowball sampling.^17^ The groups were (1) researchers, (2) hospital-based physical and occupational therapists, (3) community-based exercise trainers, (4) administrators of rehabilitation hospitals and community ABT clinics, (5) individuals living with SCI/D, and (6) advocates, funders, and policy experts. Purposive sampling^18^ was used to recruit a representative sample of six to seven males and three to four females with SCI/D.^11^,^19^,^20^

A screening questionnaire that queried the nature and duration of experience with ABT was used to determine eligibility to participate in the study. Participants were Canadian, English speaking, and either participated in, supervised, or had knowledge of ABT and SCI/D. There were no restrictions placed on years of experience with SCI/D and ABT. Written and verbal consent were obtained prior to participation in the study.

Information power, as described by Malterud et al.,^21^ was used to determine the sample size (see **Table 1**). Other literature suggests sample sizes of up to 60 participants when intergroup heterogeneity and intragroup homogeneity of focus groups exist.^22^,^23^ As such, we aimed to carry out 12 separate focus group meetings, two for each interest group, with four to five participants per group.^22^

**Table 1.** Sample size rationale using information power^21^

**Key factors**	**Justification**	**Favours small or largesample size?**
Study aim: *Narrow or Broad*?	Moderate: The study had a clearly defined but broad objective and well-defined inclusion criteria.	Moderate
Sample specificity: *Dense or Sparse*?	Moderate: The sample had well-defined inclusion criteria but a diverse group of participants.	Moderate
Established theory: *Applied or Not*?	Not: The study did not rely on a theoretical framework.	Large
Quality of dialogue: *Strong or Weak*?	Strong: The interviewer had extensive knowledge of ABT and SCI/D and experience conducting qualitative interviews.	Small
Analysis strategy: *Case or Cross-Case*?	Cross-case: A comparative analysis was performed across participant groups.	Large

Note: ABT = activity-based therapy; SCI/D = spinal cord injury or disease.

### Materials

Three separate semi-structured focus group guides were developed for this study: (1) for community-based exercise trainers and hospital physical and occupational therapists, (2) for community and hospital administrators, researchers, advocates, funders, and policy experts, and (3) for individuals living with SCI/D. The focus group guides were developed a priori based on previous work through the ABT CoP^7^-^9^,^13^ and discussions with the diverse research team representing different interest groups. Each guide contained open-ended questions that probed reasons for participating in or practicing ABT, the types of exercises and equipment they used, and the barriers and facilitators to practicing ABT (see **Table 2**).

**Table 2.** Interview questions by key interest group

**Interview questions**	**Key interest group**
Why do you choose to [use/practice] ABT?	• People with SCI/D, PT and OT, Community Trainers
OR	
Does your staff provide ABT to patients at your facility? Why/why not?	• Hospital Administrators
OR	
Why did you choose to open or manage an ABT centre in your community?	• Clinic Administrators
OR	
Skip question.	• Researchers, Advocates, Funders, and Policy Experts
Do you find ABT to be different from the therapy [you received while you were an/offered to] inpatients or outpatients in a neurorehabilitation centre?	• People with SCI/D, PT and OT, Community Trainers, Hospital/Clinic Administrators, Advocates, Funders, and Policy Experts
If yes, how so?	
OR	
Skip question.	• Researchers
What do you do in your ABT sessions [with patients/with clients]?	• People with SCI/D, PT and OT, Community Trainers, Hospital/Clinic Administrators
On average, how often do [you/your staff] [go/see your patients]?	
On average, how long are each of [your/their] sessions	
OR	
Skip question.	• Researchers, Advocates, Funders, and Policy Experts
Which exercises/activity-based exercises do you feel [you/your patients/your clients/people with SCI/D] would benefit from the most?	• All groups
AND	
Are there any exercises that you would like to [do/see clients/patients doing] in [your/their] sessions that [you/they] are not doing?	
If yes, which ones and what do you think is preventing [you/them] from doing it?	
(Probe: time, cost, need more than one therapist, equipment not available, therapist not trained?)	
Do you use equipment during your ABT sessions? If yes, what do you use?	
Which equipment do you like best/least?	
why?	
OR	
Skip question.	• Hospital/Clinic Administrators, Advocates, Funders, and Policy Experts
Which pieces of equipment do you feel benefit [you/your clients/patients] the most/least during [your/their] therapy sessions?	• People with SCI/D, PT and OT, Community Trainers, Hospital/Clinic Administrators
OR	
Which pieces of equipment do you feel people with SCI/D would benefit from the most?	• Researchers, Advocates, Funders, and Policy Experts
Is there any equipment you would like to try that you haven’t? Which ones?	• People with SCI/D, PT and OT, Community Trainers
Why haven’t you tried them?	
OR	
Is there any equipment that you would like to see patients using in their sessions?	• Hospital/Clinic Administrators
If yes, which ones and what is preventing them from using it? (Probe: don’t have it, lack of training, limited availability, multiple staff needed?) Is there any equipment you would like to purchasethat you haven’t?Which ones? Why haven’t you purchased them?	
OR	
Are there any equipment or exercises that held a lot of promise, but did not live up to your expectations or you found to be ineffective?	• Researchers
OR	
Skip question.	• Advocates, Funders, and Policy Experts
Do you experience barriers or challenges in [participating in/practicing] ABT?	• People with SCI/D, PT and OT, Community Trainers
If yes, what are your barriers or challenges to [participating in/practicing] ABT?	
OR	
Are there any barriers or challenges to implementing ABT at your institution? If yes, what are the barriers or challenges to implementing ABT?	• Hospital Administrators, Researchers
OR	
Are there any barriers or challenges to running an ABT centre and providing ABT to your clients? If yes, what are your barriers or challenges?	• Clinic Administrators
OR	
Are there any barriers to ABT practice and implementation?	• Advocates, Funders, and Policy Experts
Is there anything that helps you [participate in/in your practice] of ABT?	• People with SCI/D, PT and OT, Community Trainers
OR	
Are there any aspects of ABT that you think could be easily implemented into your [practice/institution]?	• Hospital Administrators
OR	
Are there any facilitators to running a centre?	• Clinic Administrators
OR	
Are there any facilitators to ABT practice and implementation?	• Researchers, Advocates, Funders, and Policy

*Note*: Interview questions posed (left column) according to key interest group (right column). Questions presented include the minor revisions. ABT = activity-based therapy; OT = occupational therapist; PT = physical therapist; SCI/D = spinal cord injury or disease.

### Data collection

Focus group meetings occurred between June and November 2020 over web conferencing (Zoom Video Communications) due to the COVID-19 pandemic and geographic spread of participants. Focus group meetings with each interest group lasted 30 to 80 minutes and were led by A.K., a female who identifies as a woman with 25 years of experience living with SCI/D, 7 years of experience participating in ABT, and 10 years of experience in qualitative research methods. A team member belonging to one of the interest groups (J.Z., I.M., S.M., P.A., C.R., J.L., or S.F.) also helped facilitate the focus group meetings. Following the focus group meetings, reflexivity was used to capture main ideas, impressions, and personal biases.^24^ Team meetings were held weekly to discuss completed focus group discussions, and minor revisions were made to all three focus group guides. Focus group meetings were audio-recorded, de-identified, and transcribed verbatim manually into Microsoft Word (2016).

### Data analysis

A conventional content analysis, as outlined by Hsieh and Shannon,^25^ followed by a comparative analysis of multiple perspectives of the same phenomenon (i.e., ABT), described by Lindsay,^14^ were utilized to derive meaning from the transcript data. Team members A.K. and J.S. initially read through the transcripts multiple times to immerse themselves in the data. They then proceeded to separately code three transcripts from three different interest groups using an inductive approach. A third team member (K.E.M.) met with A.K. and J.S. to discuss the initial coding list and group codes into categories of information. The remaining transcripts were then separately coded by A.K. and J.S. with additional codes added as new information emerged. Team meetings were held to discuss the coding list and organize codes into categories and preliminary themes. Microsoft Excel (2016) was used to sort codes by category and interest group for further analysis. Once all transcripts were coded, team members A.K. and K.E.M. compared transcripts within and between groups and reached consensus on emerging themes.

### Trustworthiness

Trustworthiness, a measure of validity and reliability, as outlined by Shenton^26^ was accomplished in several ways. First, the expertise of team members who performed the data collection and analysis ranged from novice to expert in ABT, SCI/D, and qualitative methods, which helped to reduce any potential bias during data interpretation. Second, investigator triangulation was established through the use of multiple team members to carry out the data collection and analysis. An audit trail was used to document this process. Third, data triangulation was achieved through the use of verbatim accounts, reflexive notes, and previous work.^7^-^9^,^13^ Finally, study findings were verified with team leads of each interest group who were part of the study team and facilitated a focus group meeting. Verbatim quotes were used to support identified themes.

## Results

Forty-eight individuals representing six key interest groups participated. The key interest groups ranged from 5 to 12 participants, with three groups (researchers, hospital therapists, and advocates, funders, and policy experts) not reaching the minimum target of eight participants. A representative sample of seven males and three females with SCI/D participated in the study. Expertise in ABT and SCI/D ranged from 0.25 to 33 years. Details of participant characteristics are presented in **Table 3**. Ten focus group meetings ranging from two to six participants were conducted along with two one-on-one interviews due to scheduling conflicts.

**Table 3.** Participant characteristics

**Characteristics**	**People with SCI/D**	**Hospital PT and OT**	**Community Exercise Trainer**	**Hospital and Community Administrator**	**Researcher**	**Advocates, Funders, and Policy Experts**
Participants (*n*)	10	6	12	8	7	5
Focus group 1 (*n*)	6	5	5	5 (Community)	3	5
Focus group 2 (*n*)	4	n/a	7	2 (Hospital)	4	n/a
Individual interview	n/a	1	n/a	1 (Hospital)	n/a	n/a
Gender (M/F)	7/3	1/5	4/8	1/7	4/3	3/2
Region of Canada (*n*)	Western (2) Prairies (2) Central (4) Atlantic (2)	Central (1) Atlantic (5)	Western (3) Prairies (5) Central (4)	Western (2) Prairies (2) Central (4)	Western (2) Central (5)	Western (2) Central (3)
Level/severity of injury (*n*) or occupation (*n*)	Cervical (8) Thoracic (1) Lumbar (1) –––––––––––––––––––– A (2) B (3) C (2) D (2) UNK INC (1)	OT (1) PT (5)	Exercise therapist (6) Kinesiologist (5)PT (1)	Community (5) Hospital (3)	Clinician scientist (2) Professor (2) Scientist (3)	Advocate (1) Funder (3) Policy expert (1)
ABT & SCI/D knowledge and/or experience(range in years)	2-13	4-30	0.25-10	1-12	1-30	5-33

*Note*: ABT = activity-based therapy; Atlantic = New Brunswick, Nova Scotia, and Prince Edward Island; Central = Ontario and Quebec; F = female; M = male; n/a = not applicable; OT = occupational therapist; Prairies = Manitoba and Saskatchewan; PT = physical therapist; SCI/D = spinal cord injury or disease; UNK INC = unknown incomplete SCI/D; Western = Alberta and British Columbia.

Six themes reflecting the challenges related to ABT practice in Canada were identified: (1) challenge of gaps in knowledge and training, (2) challenge of standardizing ABT, (3) challenge of determining the optimal timing of ABT, (4) challenge of defining, characterizing, and achieving high dosage and intensity, (5) challenge of funding ABT, and (6) challenge of measuring participation and performance in ABT. See **Table 4** for quotes by theme with Q1, Q2, etc. linking the text to the supporting quotes.

**Table 4.** Themes and quotes

**Theme 1: Challenge of gaps in knowledge and training of gaps in knowledge and training**	
Quote no.	Quote
Q1	“With that task-specific, repetitive, neuromuscular activation we can see improvements.” (P10, Community Trainer)
Q2	“A lot of the regular day-to-day activities that we do with patients are essentially activity-based therapy as well because we’re doing that mass practiced, task-specific…functional activities.” (P2, Hospital Therapist)
Q3	“I feel like a lot of people that I’ve talked to and are going to rehab, when I mention it, it’s just kind of unheard of in some clinics.” (P4, Person with SCI/D)
Q4	“We are not still clear in many of the sites what ABT is, so we are still struggling with how this is and what is conventional therapy.” (P5, Advocate)
Q5	“[I] was fascinated by the different types of things they were doing with spinal cord injuries and [it] kind of went against a lot of what I learned in college.” (P2, Community Trainer)
Q6	“I think it’s not properly trained. Not just necessarily with how to use the machine, but how to use it with our spinal cord clientele…how to be effective with it.” (P5, Hospital Therapist)
Q7	“There’s a lot of rehab therapies in the stroke population, that requires lots of repetitive training…I’m not quite sure that we are necessarily learning from that literature or borrowing their literature…I know that the two injuries have different mechanisms etc., but at the same time could we be sharing some similar methods…so that we are not just developing things independently.” (P2, Researcher)
Q8	“One of the reasons why I find these conversations super interesting is hearing about the different things that are happening at the other centres…I really appreciate knowing what else is out there so if we have folks who are interested, then for example in [city] we can send folks up to [name]’s place so they can go if that’s the kind of programming or equipment they want to have access to. So, it’s so interesting hearing about all of the other stuff that is out there.“ [P4, Community Administrator)
**Theme 2: Challenge of standardizing ABT**	
**Quote no.**	**Quote**
Q9	“At all sites, it’s not a standard protocol, all rehab sites do it and they all don’t have the same capacity [of] what they can deliver…they are not standardized from one site [to the next].” (P4, Advocate)
Q10	“One of the things that doesn’t, to my knowledge, exist right now and it could be really helpful in implementing ABT more, is the knowledge of who will benefit from what, [and] to what degree.” (P2, Hospital Administrator)
Q11	“If we’re specifically talking about repetitive activation below the level of the injury, that’s the other piece that I struggle with. Is that the patient’s activation or does passive movementqualify as ABT?...I mean, even just the piece ‘below the level of the injury’, how is that defined? Is that the neurological level of injury as defined by the ASIA Impairment Scale or is it…some other definition?” (P2, Hospital Administrator)
Q12	“To recommend what will work, what will not work, I don’t think we have enough evidence to say…Just for neuromodulation, there are so many different kinds of neuromod machines itself, so we don’t know what we’re using will work with what, so lot of unknowns out there.”(P4, Advocate)
**Quote no.**	**Quote**
Q13	“We tend to kind of think about a lot of robotic things that we do with people, but I think that people also can do a lot of home exercises that they can repeat again and again in a programmatic fashion and not necessarily using any device like FES cycling or robotic devices. And, we tend to not pay so much attention to those things probably because it’s not as sexy in terms of getting funding and no industry support. Some people want to have industry support and I think those things are really underserved or underdeveloped.” (P2, Researcher)
Q14	“Sometimes technology it’s not necessarily needed but it does provide a good help. There’s the research behind gait training is that it really doesn’t matter what form it is. Ironically enough, Lokomat came away with one of the lower scores…as far as other gait training methods, they’re all essentially identical. But it would be nicer to not have our therapists bekilling themselves every day.” (P2, Community Administrator)
Q15	“Problem with [the Lokomat] is people like using it. Participants, the clients, like using it ‘cause it feels what walking used to feel like. They see themselves; they look like how they used to walk, but it’s too passive for the most part.” (P4, Researcher)
**Theme 3: Challenge of determining the optimal timing of ABT**	
**Quote no.**	**Quote**
Q16	“I think timing since injury will also play a role here. So, if somebody has been injured for 20 years, can they go and get ABT? Are we just looking at prospectively patients who are doing ABT or…what if people with spinal cord injury are chronic patients? So, if we can deliver this to those as well, it would be helpful. So, we don’t just want to see that in future…what is available, but also people who have been injured for a long time, what is the impact of that doing ABT and if they want to do it now, what is the trajectory as well?” (P4, Advocate)
Q17	“We’re dealing with humans and they have to want to participate…And, [in] my experience…there is a group of people…who really want therapy done to them…They want to come, receive therapy so that they get better, but they don’t necessarily want to do the work that they would need to put in in order to, and the effort that they would need to do in order to make significant changes, and there’s lots of reasons for that, psychosocial reasons, cultural reasons, all kinds of things that can be implications in that.” (P2, Hospital Administrator)
Q18	“I don’t think we know as therapists how soon we’re mobilizing individuals, if it’s too soon. Because certainly following a spinal cord injury, blood pressure management is super important. We don’t want to cause tension around the cord and take blood away from the cord. So, I think we don’t have good evidence on the early stages of the timing of when to start.” (P1, Advocate)
Q19	“I feel like if it could be introduced earlier, like during your inpatient time, I feel like that’s what I would want right now to change.” (P4, Person with SCI/D)
Q20	“I think the major moment to get this vocabulary in, and this philosophy and the benefits ofABT is at our stage, right away in acute rehab to start that discussion, because this is where we see them most intensively.” (P5, Hospital Therapist)
**Theme 4: Challenge of defining, characterizing, and achieving high dosage and intensity**	
**Quote no.**	**Quote**
Q21	“We’ve got clients who are coming half an hour once a week who are some of the more functioning clients all the way up to…5 days/week for 3-4 hours/day.” (P1, Community Administrator)
Q22	“For a while I was doing 3 times a week for 3 hours, then I did 3 times a week for 2 hours. I ended up sticking with twice a week for 2 hours, which seemed to be kind of more functional for me on a recovery basis, but then I would do stuff at home as well.” (P5, Person with SCI/D)
Q23	“We do have some work in spinal cord, I think, emerging about dosing needed, but I don’t think we’re there yet to know exactly what that is for each type of injury.” (P1, Advocate)
Q24	“We definitely do see more success with more frequent and longer sessions.” (P6, Community Trainer)
Q25	“Based on what the public system can offer…we’ve seen a desire to ensure that more people are getting access to some intervention rather than a few people getting access to all of the intervention. And, so when you’re trying to create a system that’s in that way more equitable it means that everybody gets a lot less…in the ‘90s we were seeing 100 patients a year…now we’re up to 300. So, that means 200 additional people a year are getting access, a bit of access, to what we have to offer.” (P2, Hospital Administrator)
Q26	“Our full-fledged mandate is to train our clients to gain independence to get them home. So, we definitely have stress, we hear about our waiting list from the hospital, our job is to go fast.The challenges of the health care system…if we have waiting time at the acute hospital, we’re going to be charged. So, we have that pressure that we can’t take extra time…as clinicians, we shouldn’t be necessarily only thinking of that, but we do have that pressure of discharge and length of stay that is for sure present. So, then we go towards our traditional interventions without thinking outside the box, because we know that’s our primary goal here is to create, to foster that functional independence, to get the patient home.” (P5, Hospital Therapist)
Q27	“Inpatient facilities…have a very finite amount of time and resources to get that client out into the community…So, when we talk about rehab, we’re talking about 2-5 hours a day. The inpatient care setting in the public health care system just has no chance of doing something like that with any person.” (P2, Community Administrator)
Q28	“Therapy’s not cheap. A lot of clients are paying out of their own pockets, so unfortunately the finances are a really big determining factor for peoples’ frequency here.” (P6, Community Trainer)
Q29	“We’re very reliant on volunteers. Without volunteers, our FES program just wouldn’t exist, I’ll flat out say that. We need them to be able to run it the way we do.” (P5, Community Trainer)
Q30	“You need to be able to measure how intense the workout is and how hard the person’s working. So, I think cardiovascular measures correlated to distances and repetitions and weights…understanding is that heart rate elevating? Are they breathing heavily? Are they actually working hard? How much effort are they putting in? And then, what are they actually achieving with that effort? How many reps are they doing? How far are they going? How much weight are they lifting? How many steps are they taking? Whatever those measurements are to be able to paint that picture of how hard somebody’s working and how that correlates to their improvement in function.” (P5, Advocate)
Q31	“I would make it more around the nervous system than the cardiovascular system. So, the sensory and motor inputs that are going to help with whether it be central pattern generation or reciprocal movements that are trying to look for patterning.” (P5, Researcher)
**Theme 5: Challenge of funding ABT**	
**Quote no.**	**Quote**
Q32	“Time and resources and funding. Those are the main ones, always. I’m sure that’s not news to anyone and this is coming from one of the centres that probably has more of all of those things, and yet it still is a challenge for us, so it’s certainly a challenge for everyone else in the country, I’m sure.” (P2, Hospital Therapist)
Q33	“This is a systemic problem. I think our length of stays are shortening, our outpatient therapies are shrinking, there’s no home care therapy without paying for it out of pocket, over 80% of people who have spinal cord injuries in this province do not have means of third-party funding to afford therapies if they’re out of their own pocket, and that there is no clear systemic process to achieve long-term ABT the way the system presents itself today.” (P3, Advocate)
Q34	“Funding structure in (province) is very different than (province) and (province) and we’re not as much opportunity for funding [for equipment] in the hospital, especially for rehab, and there is the big foundation that does offer funding for the hospital in general, but rehab is usually lower on the list unfortunately.” (P4, Hospital Therapist)
Q35	“I like the MyndMove… I was like, ‘This is it! This is the thing!’ And, then they made it not available to us. We can’t use it. We have to go through this really weird funding model and I think that’s terrible. You would think that that would be something when you’ve seen such wonderful results from it that it should be accessible to the patients individually; shouldn’t cost an arm and leg, should be accessible to us as clinicians, shouldn’t have to be a big research centre to get it.” (P1, Hospital Therapist)
Q36	“The first barrier to think about is just process. So, if we were going to implement a new approach or a new therapy, we would just need to make sure we have a good change management process, like how it’s going to change the way and the journey of the patient in their rehab stay here. So that would be more of a process issue to look at what are we currently doing and how do we integrate this in a meaningful way. Financially and sort of structurally; if we’re looking at putting any additional space, equipment needs, you just haveto really look at the space that you have, what could go into it as it is or what would need to be modified if you needed more square footage.” (P3, Hospital Administrator)
Q37	“Funding tends to be one of the biggest challenges right here in getting the piece of equipment.” (P5, Community Administrator)
Q38	“It’s not as readily available to people, particularly through government programs. It’s facilitated all through fee-for-service at this point in this province.” (P3, Advocate)
Q39	“Us being a charity…we’ve been able to get a lot of our equipment donated. So, while a lot of people don’t want to fund therapy costs for individual clients, I think we have about $500,000 worth of equipment in our facility that’s all been bought through donations to (facility) for those specific pieces. So, I think that’s helped a lot, being able to get a lot of that equipment.” (P1, Community Trainer)
**Quote no.**	**Quote**
Q40	“If the ultimate goal is to get this into rehabilitation, I think you need a cost-benefit analysis…you need to demonstrate the cost outcome and the benefits of investing into ABT. I also think that there needs to be a correlation to service, particularly attendant services. Will ABT allow people to be more independent, to be less reliant on service? I think it’s a very important indicator for governments and other decision bodies to consider new funding models. I think this requires a business case. I think the evidence of the efficacy of the…therapies in itself is not enough to be able to look at ABT as a standardized method of rehabilitation in all government funded programs.” (P3, Advocate)
Q41	“One of the difficulties with economic evaluations with ABT research is sometimes the benefits are not necessarily highlighted or revealed in the way that we…find valuable. There may be some difference when we look at cost health care utilization, but in terms of looking at health-related quality of life it’s uncertain at this point whether using ABT or not, whether we’d be able to notice any difference at all looking at those types of outcomes. In particular, quality of life, longevity of life, those are sort of the gold standard outcomes that are used in economic evaluations and knowing if we can find a difference with the use of ABT; it’s an area that needs to be explored.” (P3, Researcher)
Q42	“The quality of the evidence in terms of trying to determine cost effectiveness is just not there at all…that’s because those [high tech] ones tend to be linked to the clinical trial, and so it’s always going to look horrible short term, and it’s many of these [studies] don’t explore the long-term consequences, where you’re expecting the savings to come back…the ones that are done right now don’t look good ‘cause its short term. It’s expensive, it can’t overcome the price tag of the device.” (P3, Researcher)
**Theme 6: Challenge of measuring participation and performance in ABT**	
**Quote no.**	**Quote**
Q43	“If you want to know whether how well ABT works, or if it works better than other things, or if it compliments other things, so we need to be able to know what was delivered before we can know whether or not it worked.” (P1, Researcher)
Q44	“We don’t have good tools. We can’t even measure…what we’re delivering. We will measure activity in terms of time that they spent typically in therapy…we talk about what we did and how long the session was, but…we didn’t have the technology to measure the dose…we don’t have all the building blocks needed to actually move to providing this in a structured, standardized format with goals in mind.” (P1, Advocate)
Q45	“I think one of the main barriers or challenges is that there hasn’t been standardized outcomes, so we’re all kind of measuring as much as we can and I think…very often it’s at the end of your study that you realize you might’ve benefitted from tracking something else. So, if we had standardization of outcomes that were evidence-based that we all agreed upon, that would be great.” (P4, Researcher)
Q46	“Tracking over time will be very important. I know in rehab you use FIM, but in ABT I don’t know if those will be relevant or not.” (P4, Advocate)
Q47	“Are we calculating the number of steps they do all day? Or is it just the 45 min to an hour with me?...I know that my little hour with the patient within 23 hours of their day is not much so I’m quite aware that…what I do with the client is just to encourage them toward something, but what they do the entire day has to count down the line. All the activities that they’re doing in their room, that they do with their family, with the nurses, somehow and that’s a big challenge, but somehow it has to count somewhere ‘cause I think they develop a lot by themselves, and then just reinforce some of the things that they’re learning with me.” (P5, Hospital Therapist)
Q48	“The tool we use I always criticize it for being not sensitive enough to pick up pretty good functional movement. So, one example of ours, like we’ve shown through research that 94% of our people have increased movement below their level of injury. One of the people who was in the 6% who didn’t was able to ride a bike, like literally start it, stop it, but that’s not a functional movement so you kind of feel like that person made huge progress, but it’s not a functional movement as defined by a chart or by a developmental activity scale.” (P2, Community Administrator)
Q49	“When we talking about prevention, tracking outcomes is so difficult. … And we often times have challenges with that and then whatever outcomes we generate from that to try to convince other people that it works, and especially that’s why you have to implement that clinically. They don’t see it as convincing because people are so used to seeing, okay…the bad stuff got better. They want to see, just like pressure ulcers, they want to see the pressure ulcer improving, but they don’t want to see how you prevent it, right? So, it’s really…difficult, but at the same time to me it’s such an important aspect of ABT.” (P2, Researcher)
Q50	“Whatever those measurements are to be able to paint that picture of how hard somebody’s working and how that correlates to their improvement in function and then to tie that into…how much is that reducing their cost of care? How much more independence are they getting? And, how much less reliant are they on other sources of care provision?” (P5, Advocate)

Although all groups reported challenges across each theme, the comparative analysis revealed that some challenges were emphasized by one or a few groups. Hospital and community clinicians described the lack of quality education and training (theme 1) and achieving a high dosage and intensity in ABT as key challenges (theme 4). Researchers and advocates, funders, and policy experts emphasized the challenge of developing standardized guidelines (theme 2) and performing economic analyses to measure the impact of ABT programs (theme 6). With respect to developing guidelines (theme 2), groups also expressed contrasting views on the benefits of some high-technology equipment, with clinicians and people living with SCI/D favouring them over researchers. Persons with SCI/D specifically struggled with the high cost and lack of access to ABT programs and equipment, whereas hospital and community administrators were similarly challenged with securing the funding needed to support the high demands of an ABT program (theme 5).

### Challenge of gaps in knowledge and training

Participants across all groups acknowledged that there was a general lack of knowledge and understanding about ABT within their peer groups. Some participant groups (people with SCI/D, community administrators, and trainers) demonstrated a clear understanding of ABT and its principles when describing ABT practice or engaging in ABT by using key terminology such as targeting muscles “below level of injury,” “repetition,” and “task-specific movement” (Q1). A few hospital therapists pointed out that once they learned about the definition of ABT they recognized that they actually practiced ABT to a much greater extent than they realized (Q2). Individuals with SCI/D did mention, however, that many of their peers were unfamiliar with ABT when they spoke to them about it (Q3). Advocates, funders, and policy experts, researchers, and hospital administrators agreed, believing that many of their colleagues lacked clarity on how ABT was defined, what it encompassed, and how it differed from conventional therapy (Q4).

Community trainers spoke about how their postsecondary learnings about SCI/D, its prognosis, and therapies were inaccurate and opposite to what they observed in practice (Q5). Hospital therapists described being inadequately trained with specific types of equipment, like electrical stimulation devices, which led to fears and lack of use in practice (Q6). Researchers, community trainers, and hospital and community administrators likewise talked about the importance of training on the appropriate use of equipment and adapting the way equipment is used to maximize the potential benefits to people with SCI/D who participate in ABT.

…any equipment being misused or not being used to its potential. It really is matching what the equipment is…capable of because we often don’t have the understanding of what to do at the right time…I’ve seen people really take advantage of what an exoskeleton can offer, and then I’ve also seen people not and it’s really, they’re using them for different reasons and different purposes and it really is matching them to the individual. I think it’s the how equipment is used…[it can be] disappointing, but in the right hands can be really exciting. (P5, Researcher)

Researchers and advocates, funders, and policy experts discussed the benefit of learning about ABT practice from other populations, such as stroke, in order to apply learnings to SCI/D (Q7). All groups stressed the importance of collaboration and knowledge sharing—between research and clinical practice, and across community and rehab centres—as a key to optimizing and advancing practice (Q8).

### Challenge of standardizing ABT

According to researchers and advocates, funders, and policy experts, there are currently no standardized guidelines for ABT (Q9), and the need for developing an evidence-based, standardized approach to ABT practice was emphasized. Standardized guidelines could provide information for clinicians regarding the types of ABT activities, exercises, and techniques that are most effective in promoting recovery based on injury characteristics. Some groups (advocates, funders, and policy experts, researchers, hospital therapists, and hospital administrators) reported a dearth of evidence in this area (Q10), which limits the ability to develop standardized guidelines. Researchers also described the challenge of standardizing ABT due to the variety of activities and exercises available and ways of characterizing them.

It would also be the actual therapeutic activities, in addition to the outcomes, that are even more challenging to put some standardization around what is actually being done. There’re so many things that might be done and ways to characterize them that developing a system for that that would be standardized is very challenging. (P5, Researcher)

Researchers and hospital administrators pointed out that without consensus on the definition and parameters of ABT, we may not be speaking the same language when we talk about ABT (Q11). Advocates, funders, and policy experts further questioned the relevance of defining the therapy provided as being ABT or conventional therapy and suggested the focus should instead be on determining the ideal mix of therapies needed to optimize recovery.

Kind of a combination of interventions and some of it is maybe possibly labeled under the activity of ABT and some are not, but maybe that’s okay. Do we need a definitive defining therapy intervention that we’re calling something very specifically? Or is it a mix or combination of interventions that is getting you the actual result regardless whether it’s called ABT or something else? A smash between a combination of things is going to actually get you the functional recovery that people are seeking. (P3, Advocate)

Equipment and technology were discussed at great length by the various groups suggesting standardized guidelines should include recommendations on appropriate use of technology in ABT (Q12). Researchers explained that although a great amount of research has focused on high-tech, costly robotics like the Lokomat, the equipment has not demonstrated superior benefits to justify their costs and reflected that further research should consider exploring the benefits of other exercises that don’t require high-tech devices (Q13). Interestingly, hospital and community administrators, therapists, and trainers agreed that certain high-tech equipment was a disappointment, but it may be valued because it reduces the strain of manual labour and preserves the health of therapists and trainers (Q14). Individuals with SCI/D also tended to be enamored by the high-tech equipment they heard about or used; this was something researchers also recognized through their clinical trials (Q15). The diverse views expressed across groups highlight the challenge of developing standardized guidelines to ABT practice and technology use.

### Challenge of determining the optimal timing of ABT

Participants indicated that little was known about the optimal timing for engagement in ABT. For example, one advocate questioned if and how time since injury influenced the potential to benefit from ABT (Q16). Although evidence on the optimal timing of ABT is lacking, participants identified numerous factors that impacted readiness to engage in ABT. Advocates, funders, and policy experts, researchers, and hospital and community administrators spoke about motivation being a key factor to participation in ABT. Levels of motivation may be influenced by age at the time of injury, time postinjury, psychosocial, cultural, and personal factors, and the need to focus on other goals of rehabilitation (Q17).

Aside from level of motivation, hospital administrators were concerned that individuals with SCI/D were not physically capable or emotionally ready to participate in intensive therapy early postinjury as they were still coming to terms with their injury. Unfortunately, they often found that the timing was misaligned; readiness to participate often coincided with discharge from inpatient rehab.

When we think of the timing of when everything happens…everything’s shifted to start earlier so people come to us within a week of their injury, which I think is the right thing in a lot of ways…But, are they physically and emotionally ready to participate fully? A lot of times, no. So, they’re still a week within their surgery and we’re trying to challenge them to roll and stand and turn and all these things, but then when they’re often in the prime of both their neurological recovery and their ability to engage and participate, that’s when they’re being discharged. (P1, Hospital Administrator)

An advocate with a background in physical therapy also expressed concerns regarding the safety of participating in ABT in the acute stages of injury while the body was still healing from the initial trauma (Q18). However, most participants with SCI/D, including a few advocates with SCI/D, stated that they would have appreciated the opportunity to experience ABT earlier postinjury (Q19). One hospital therapist agreed stating that early rehabilitation was the perfect time to introduce ABT because of the resources and therapy time available to them (Q20).

### Challenge of defining, characterizing, and achieving high dosage and intensity

Participants identified a lack of clarity concerning the dosage and intensity of ABT as a challenge. In hospital-based rehabilitation, the dosage (i.e., frequency and duration of sessions) is defined by the structure of service delivery, whereas in the community, dosage can vary (Q21). Individuals with SCI/D spoke about initially adjusting their ABT program until they found their optimal dosage (Q22). Regardless, advocates, funders, and policy experts, researchers, and community administrators stated that there is a lack of, and need for, evidence on optimal dosing (Q23); however, higher dosages were perceived to lead to better outcomes (Q24).

Achieving a high dosage is challenging in both the hospital and community settings for several reasons. Hospitals are challenged with trying to provide equitable access to care for all individuals who experience SCI/D. Compared to the past, this translates to more individuals receiving some therapy rather than a few individuals receiving a high amount of therapy (Q25). Researchers and hospital therapists and administrators described the pressures of trying to balance neurorecovery with functional independence and readiness for discharge within the limited time they have with their patients (Q26).

Community administrators acknowledged that due to constraints within a publicly funded healthcare system, hospital therapists would be unable to offer their patients a higher dosage; this is something the community is able to provide to clients who desire it (Q27). Nonetheless, there are still a number of challenges that prevent individuals with SCI/D from reaching a high dosage in community. Cost of therapy and lack of third-party coverage are main reasons for individuals not participating in ABT more often (Q28). Other factors reported by community trainers and persons with SCI/D that may affect an individual’s ability to sustain a high dosage of ABT are other competing interests such as work, family obligations, or other therapies. The necessity of support staff, trainees, students, and volunteers were voiced strongly by hospital therapists, community trainers, hospital and community administrators, and persons with SCI/D without whom they wouldn’t be able to provide ABT to the number of individuals with SCI/D to the extent that they do (Q29). Similar to timing and readiness, motivation ties in here as well since individuals need to not only be willing to participate in ABT but also be willing to exercise at an intensity needed to achieve neurorecovery.

Exercise isn’t for a lot of people, and this kind of exercise takes a lot of work, and a lot of effort; a lot of dedication to see the benefits. And, so if you’re not willing to put that in, and if you’re not willing to work that hard, cause I mean like we have clients who come here, this is their social outing. They come here once a week and yeah, they try, but they’re not really in it. They’re not gung-ho and really pushing themselves and everything. (P3, Community Administrator)

Participants often used the words “dosage” and “intensity” interchangeably, suggesting a lack of clarity on the differences between the two terms. Participants, even within the same group, had differing views on how intensity is gauged, characterized, and measured when describing ABT. A researcher and an advocate with a physical therapy background suggested focusing on the cardiovascular system as a way to measure exercise intensity, while another advocate suggested including other parameters, such as distance, number of repetitions, and weight (Q30). In contrast, another researcher thought the emphasis should be on the nervous system (Q31).

### Challenge of funding ABT

Hospital- and community-based rehabilitation settings are challenged with acquiring the necessary funds to provide ABT to people with SCI/D. One hospital therapist identified funding as one of the top three challenges they faced (Q32). An advocate believed the funding challenge to be a systemic issue reaching across the continuum of care (Q33). Existing differences in funding structures across provinces, as mentioned by another therapist, pose additional challenges and create inequities in access to ABT (Q34). In addition, there is the challenge of accessing beneficial technology due to complex funding models (Q35). Hospital administrators consider financial barriers to implementing ABT as a component of a larger issue that looks at the process as a whole and considers elements such as space to accommodate new equipment (Q36).

Unlike hospitals, which are publicly funded, community-based ABT clinics are privately owned and rely on fee for service to operate. Community trainers and administrators describe the struggle of accessing funds for equipment (Q37). Hospital therapists and advocates, funders, and policy experts acknowledge the importance of having ABT available in the community for individuals postrehabilitation and believe the clinics should be able to access supports through the government (Q38). In response, community clinics have applied for charity status in order to receive donations, host fundraising events, apply for grants, and maintain affordability for clients (Q39). Some community clinics received equipment donations, which was helpful to some extent but not necessarily ideal.

Standing frame, it was donated to us by one of our clients who’s a rather slight framed girl, so definitely sized for somebody smaller. Whereas, I know there are a tonne of different types, different models from the same company that might be more accommodating of larger clients. So, at the same time I think, this one, which is more of a base model, is still over $4,000, so there’s a bit of a financial constraint when it comes to that thing, right? To have your clinic suited up with one that perfectly fits any given client is not exactly financially feasible. (P6, Community Trainer)

Researchers and advocates, funders, and policy experts stressed the need to conduct economic evaluations to determine the cost-benefit analysis, attract government funding, and support implementation of ABT in various settings (Q40). However, they also recognized the challenges associated with these types of evaluations in relation to ABT as it may be difficult to demonstrate an improvement in health-related quality of life or longevity of life—the accepted gold standards (Q41). Particularly when evaluating cost-effectiveness of high-technology equipment, research evidence in this area is poor (Q42).

### Challenge of measuring participation and performance in ABT

Participants across all groups discussed the importance of having tools that can measure participation in and outcomes of ABT (Q43). Researchers and advocates, funders, and policy experts reported that currently there are no suitable tools to measure ABT dose (Q44) and no standardized approach to the collection of outcomes (Q45). Researchers, hospital and community administrators, hospital therapists, and community trainers all expressed varying degrees of dissatisfaction with some of the existing standardized tools they used to track performance in ABT. One advocate questioned whether some of the standardized tools used in rehabilitation to measure performance would be applicable to ABT (Q46). Hospital therapists confirmed that there were validated tools to measure function that were not being used consistently by clinicians. In addition, hospital therapists stressed the need to track therapy occurring outside of their one-on-one sessions to get a full understanding of an individual’s activity (Q47). One community administrator explained that some improvements individuals make don’t get captured on any tools they use (Q48). Researchers, hospital therapists, community administrators, and trainers all criticized the lack of sensitivity of existing tools, which they felt were incapable of measuring small increments of change in function over time.

Do we have the right tools that will sensitively pick up things or changes?…[D]o we have better ways to track other than neurological outcomes, which are more sensitive, and yet still can be applied easily? If you look at the ISNCSCI group in Europe then I think they do a wonderful job with electrophysiology outcomes, but can’t do that on everyone every time, so what else can we do that will be a much better tool than something like motor score or neurosensory score, because those are just so crude. (P2, Researcher)

In addition to measures of function, researchers and advocates, funders, and policy experts mentioned a few other outcomes they thought were important, yet challenging, to consider, such as secondary health complications to determine whether ABT prevents bowel, bladder, pain, or skin-related issues (Q49). Advocates, funders, and policy experts also suggested tracking long-term quality of life outcomes such as return to work, mobility device use, independence in activities of daily living, reliance on support providers, and community participation (Q50).

## Discussion

This study explored the challenges related to ABT practice and implementation in Canada from the perspective of researchers, hospital-based physical and occupational therapists, community-based exercise trainers, administrators of rehabilitation hospitals and community ABT clinics, individuals living with SCI/D, and advocates, funders, and policy experts. Although there was agreement among groups regarding challenges to ABT practice, there were challenges that were emphasized by one or a few groups. Individuals with SCI/D highlighted the cost and access to ABT and equipment as particular challenges. The minimal and sometimes inaccurate education and training in ABT and associated technologies were identified as challenges for hospital and community clinicians. Researchers, hospital and community administrators, and advocates, funders, and policy experts emphasized the lack of evidence to develop guidelines, optimize dosing, and determine cost-benefit analysis in ABT as key challenges.

The challenges identified in this study validate and coincide with four of the eight priorities for ABT research and care identified by the Canadian ABT CoP: (1) track engagement in ABT activities across the continuum of care, (2) develop and implement best practice recommendations for ABT, (3) study optimal timing, methods, and dose of ABT to promote desired outcomes, and (4) educate clinicians from across the continuum of care about ABT.^13^,^27^ The evaluation of challenges or barriers to ABT practice mark a key step in the process of translating knowledge into practice as described by the Knowledge to Action Framework.^28^ Most groups in this study identified the lack of knowledge and clarity in how ABT is defined and characterized as a significant challenge to ABT practice. At least four definitions have been reported in the literature.^1^,^4^,^29^,^30^ Definitions of ABT vary in their focus (i.e., function vs. neurorecovery) and target (above and below level of injury vs. only below level of injury). Reaching international consensus on the definition of ABT may facilitate communication across disciplines and settings (e.g., research, health administration, hospital-based rehabilitation, community clinics) and prevent confusion about which therapeutic activities are and are not considered ABT. Lack of knowledge, education, and training in ABT among therapists and exercise trainers suggests that teachings should be incorporated into entry-to-practice and postgraduate curriculums. On-the-job training in ABT through in-services and mentorship by experienced colleagues may also increase knowledge and competence in delivering ABT.

Developing standardized ABT guidelines is essential in order to guide clinicians in optimizing neurorecovery for their patients and clients. To accomplish this, we need to know the best time to provide ABT, the effectiveness of ABT activities and technologies, and the optimal dosing and intensity.^31^ Preclinical data suggest there is a small window of opportunity early post-SCI to maximize recovery, provided inflammatory responses are controlled, after which time there is no effect.^32^ However, studies have demonstrated improvements in trunk, upper and lower extremity motor function, cardiovascular fitness, and thermoregulation in individuals with complete and incomplete chronic SCI using transcutaneous electrical spinal cord stimulation alone or combined with overground gait training or pharmacological treatment, or a multimodal ABT program.^6^,^33^-^39^ The Neurorecovery Network has developed guidelines specific to locomotor training that have been implemented across several centers in the United States,^40^ yet no such guidelines exist for other interventions, such as functional electrical stimulation ergometer training. Jones et al.^41^ reports on a set of guidelines developed by a community-based ABT clinic in the United States where individuals transition through five phases of recovery; each phase is associated with specific interventions and techniques to promote neurorecovery. The authors acknowledge the lack of clarity pertaining to optimal dosing and intensity, yet they maintain that a higher dosage and intensity of therapy is needed than what is currently available. The literature reports a dosage of ABT ranging from 90 minutes to 3 hours per day, three to five days per week.^4^,^41^-^43^

Hospital and community settings are both uniquely challenged with striving to achieve a high dosage and intensity of therapy among their patients and clients. Studies in two Canadian inpatient rehabilitation centres found the intensity of cardiovascular training and number of movement repetitions in the upper and lower extremities per therapy session to be insufficient to result in neurological improvements.^44^,^45^ Cost-effective ways to address these challenges are needed. One study demonstrated how the use of technology (i.e., a treadmill) increased the number of steps taken in a 1-hour locomotor training session in comparison to gait training performed overground, which led to greater improvements in walking ability.^46^ Another study found inpatients had potentially 4 hours of time available outside of their regular therapy sessions to participate in additional therapy.^47^ Where the nontherapy time is currently spent in sedentary leisure activities, individuals could instead use it to focus on independent muscle strengthening exercises above the level of injury and cardiovascular training, which would allow session times with their therapist to be devoted to ABT-related activities targeting muscles below the level of injury.

Appropriate tools that are capable of tracking the details of an ABT session, and sensitive enough to measure change in function over time, are needed and will allow us to collect the necessary data to answer many of the questions posed above. Ongoing work with the ABT CoP has explored the types of ABT activities and parameters to include in an ABT tracking tool.^15^,^48^ The Neuromuscular Recovery Scale, developed by the Neurorecovery Network for their locomotor training program, is a discriminative tool that has demonstrated responsiveness.^42^,^49^,^50^ Further research should consider evaluating existing outcome measures to determine suitability for ABT and developing a standardized approach to collecting outcome data.

Funding appears to be a chronic and systemic issue that all groups identified in ways that were applicable to them. Suitable ABT tracking tools and outcome measures will facilitate economic evaluations that may support an increase in government funding and insurance coverage for ABT across the continuum of care after SCI/D. Unfortunately, few economic evaluations of ABT for SCI/D rehabilitation have been produced. One study explored the economic benefits of locomotor training and found it reduced long-term healthcare costs associated with rehospitalization.^12^ Another study evaluated the cost-effectiveness of overground robotic locomotor training in comparison to conventional locomotor training for people with SCI; it found overground robotic locomotor training to be more cost-effective for people with complete injuries, whereas conventional locomotor training was more cost-effective for people with incomplete injuries.^51^ A recent environmental scan of studies targeting neurorecovery in people with SCI found only two of the 73 studies reported on economic factors.^1^ Inclusion of economic evaluations in future ABT research and clinical projects is needed.

### Limitations

Biases inherent in qualitative research and focus group meetings were considered in this study, and measures were taken to reduce these through efforts to ensure trustworthiness. As these meetings occurred early in the COVID-19 pandemic, web conferencing was used, which permitted geographic diversity of participants yet may have compromised the quality of data collected due to participants’ lack of comfort and familiarity with virtual platforms. Interviewer bias,^52^ social desirability bias,^53^ and conformity bias^54^ were lessened due to the experience of the interviewer (A.K.) in creating a rapport with study participants and allowing each to voice their opinion freely. Three participant groups (researchers, hospital-based physical and occupational therapists, and advocates, funders, and policy experts) failed to reach their recruitment target. There are few individuals in Canada who identify with one of these three groups and have experience with ABT and SCI/D; hence, it was not surprising to find recruitment of these three groups challenging. In addition, the COVID-19 pandemic posed a strain on the hospital system, with many staff being redeployed to other areas and researchers having to manage labs and projects that were put on hold. This made it particularly challenging to recruit hospital therapists and researchers. Nevertheless, we believe that within group diversity of participants provided sufficient variance in perspectives to minimize the impact.

## Conclusion

We are recognizing that rehabilitation is no longer a short-term venture for individuals who sustain SCI/D. ABT may provide individuals with the opportunity to continually improve function, independence, and overall quality of life. Establishing a system that can ensure all individuals with SCI/D have the ability to access and participate in ongoing therapy once they are discharged from rehabilitation is needed. This study identified challenges that currently limit ABT practice and implementation in Canada. Future research should explore strategies to address these challenges to ABT practice to support successful implementation of ABT in Canada.
